# HIV transmitting mononuclear phagocytes; integrating the old and new

**DOI:** 10.1038/s41385-022-00492-0

**Published:** 2022-02-16

**Authors:** Erica E. Vine, Jake W. Rhodes, Freja A. Warner van Dijk, Scott N. Byrne, Kirstie M. Bertram, Anthony L. Cunningham, Andrew N. Harman

**Affiliations:** 1 Centre for Virus Research, The Westmead Institute for Medical Research, Westmead, NSW Australia; 2 Faculty of Medicine and Health, School of Medical Sciences, The University of Sydney, Westmead, NSW Australia; 3 Sydney Infectious Diseases, Faculty of Medicine and Health, The University of Sydney, Westmead, NSW Australia; 4 Centre for Immunology and Allergy Research, The Westmead Institute for Medical Research, Westmead, NSW Australia

## Abstract

In tissue, mononuclear phagocytes (MNP) are comprised of Langerhans cells, dendritic cells, macrophages and monocyte-derived cells. They are the first immune cells to encounter HIV during transmission and transmit the virus to CD4 T cells as a consequence of their antigen presenting cell function. To understand the role these cells play in transmission, their phenotypic and functional characterisation is important. With advancements in high parameter single cell technologies, new MNPs subsets are continuously being discovered and their definition and classification is in a state of flux. This has important implications for our knowledge of HIV transmission, which requires a deeper understanding to design effective vaccines and better blocking strategies. Here we review the historical research of the role MNPs play in HIV transmission up to the present day and revaluate these studies in the context of our most recent understandings of the MNP system.

## Introduction

2021 marked the 40th anniversary of the first official report about AIDS and 35–43 million people have lost their lives to this disease. Despite this enduring pandemic, there is still no cure or vaccine for Human Immunodeficiency Virus (HIV). The introduction of antiretroviral therapies (ART) has improved patient outcomes and reduced the consequences of HIV infection from a terminal to a chronic illness.^1^ ART can reduce serum virus levels to undetectable levels and virtually eliminate the risk of transmission from ART-treated HIV^+^ individuals.^2^ However, treatment is still lifelong^3^ and HIV remains a substantial burden to the infected individual as well as national healthcare systems. Unfortunately, with ~16 million infected individuals worldwide not receiving ART, and the minimal use of pre-exposure prophylaxis (PrEP) in high-risk individuals,^2,3^ transmission rates remain stable. Therefore, the development of a cure and vaccine is still vital. In the meantime, an effective and fast-acting topical microbicide could be used on a per need basis. These could be incorporated into slow-release vaginal rings, lubricants, or a hygiene douche, complementing current PrEP regimes.^4,5^ However, designing effective prevention strategies requires an accurate and comprehensive understanding of the early events underlying HIV transmission.

Sexual transmission across the human genital and anorectal (anogenital) mucosa is now the predominant route of HIV transmission, but the early transmission events and the immune cells involved remains under discussion.^6–8^ These transmission sites differ anatomically, physiologically and immunologically^7–12^ but once the physical barriers of these tissues are breached, pathogens encounter a range of mucosal cell types that participate in HIV transmission, including sub-epithelial mucosal fibroblasts^13–15^ and the cellular immune systems first line of defence, mononuclear phagocytes (MNP). MNPs are immune sentinels which bind pathogens via an array of cell surface receptors triggering an immune response. In the case of HIV these cells are actively involved in transmission, disseminating virus to CD4 T cells as a consequence of their antigen presenting cell (APC) function. MNPs are therefore potential key targets for blocking HIV transmission at mucosal sites and of key importance for vaccine design.

Here we will review role MNPs play in transmitting HIV in the tissues where sexual transmission occurs. In addition to providing a historical perspective, we will review recent advances in our understanding of the specific subsets of MNPs that transmit HIV as well as highlighting gaps in the literature that may impact on our understanding of early transmission events.

## The evolving mononuclear phagocyte system

MNPs are a family of phagocytic cells, traditionally defined as macrophages, monocytes and dendritic cells (DC). Macrophages were the first MNP to be discovered by Élie Metchnikoff in 1884 for which he received the Nobel prize.^16^ In the 1920’s, monocytes were proposed to be precursors to macrophages^17,18^ giving rise the idea of a multicellular mononuclear phagocyte system. Two years after the discovery of macrophages, Langerhans cells (LC) were discovered by Paul Langerhans in 1884.^19^ However, these were first thought to be nerve cells, due to their dendritic appearance and it was not until 1973 that Inga Silberberg showed that they played a role in immunity.^20^ Also in the 1970s, Nobel laureate Ralph Steinman discovered a novel cell type that did not look like macrophages and did not easily mediate endocytosis which he named DCs.^21,22^

Since these early discoveries, multiple subsets of MNPs have been defined which have historically been classed based on their functional properties. **Dendritic cells (DC)** are potent APCs and function to sample pathogens and commensal microflora in tissue and then migrate to lymph nodes to present them to CD4 T cells via MHC-II and drive immune activation or immune tolerance. As **LCs** perform the same function as DCs and also have dendritic processes they have traditionally been considered a subset of these cells. **Macrophages** are weak APCs but play a key role in innate immunity by phagocytosing pathogens at the site of exposure and in maintaining tissue homeostasis. **Monocytes** are a population of cells in blood that migrate into tissue where they differentiate into effector DCs or macrophages based on the cytokine environment they encounter. It is of note that these monocyte-derived DCs (MDDC) and macrophages (MDM) differ to conventional DCs and macrophages.

The advent of high parameter single cell technologies (especially RNA seq) has allowed for more robust ontological phenotyping leading to alternative classifications. Two key HIV target MNPs are of point here. Firstly, LCs have been shown to be derived from the yolk sac during embryogenesis similar to macrophages, as opposed to DCs which are derived from bone marrow derived progenitors. Thus, they can be thought of as either DCs or macrophages. For this reason, LCs are now best defined as their own class of MNP. Secondly, the CD14^+^ tissue compartment, which has been undergoing an ongoing redefinition over the last decade. As will be discussed in detail later, tissue CD14^+^ cells were originally believed to consist of tissue-resident yolk sac derived macrophage (defined by their autofluorescent properties) and a type of conventional DC (defined by their lack of autofluorescence).^23^ The DC component was later redefined as MDMs^24^ and more recently shown to consist of a heterologous population of MDMs and MDDCs.^7^ More recently, this compartment has also been shown to contain a new defined bona fide bone marrow derived DC population named DC3.^25–28^

Therefore, the MNP system is in reality a spectrum of cells derived from a variety of distinct precursors that perform a range of functions including pathogen detection and clearance, antigen presentation and tissue homeostasis. The full range of currently defined human MNPs is illustrated in Table 1.

**Table 1:**
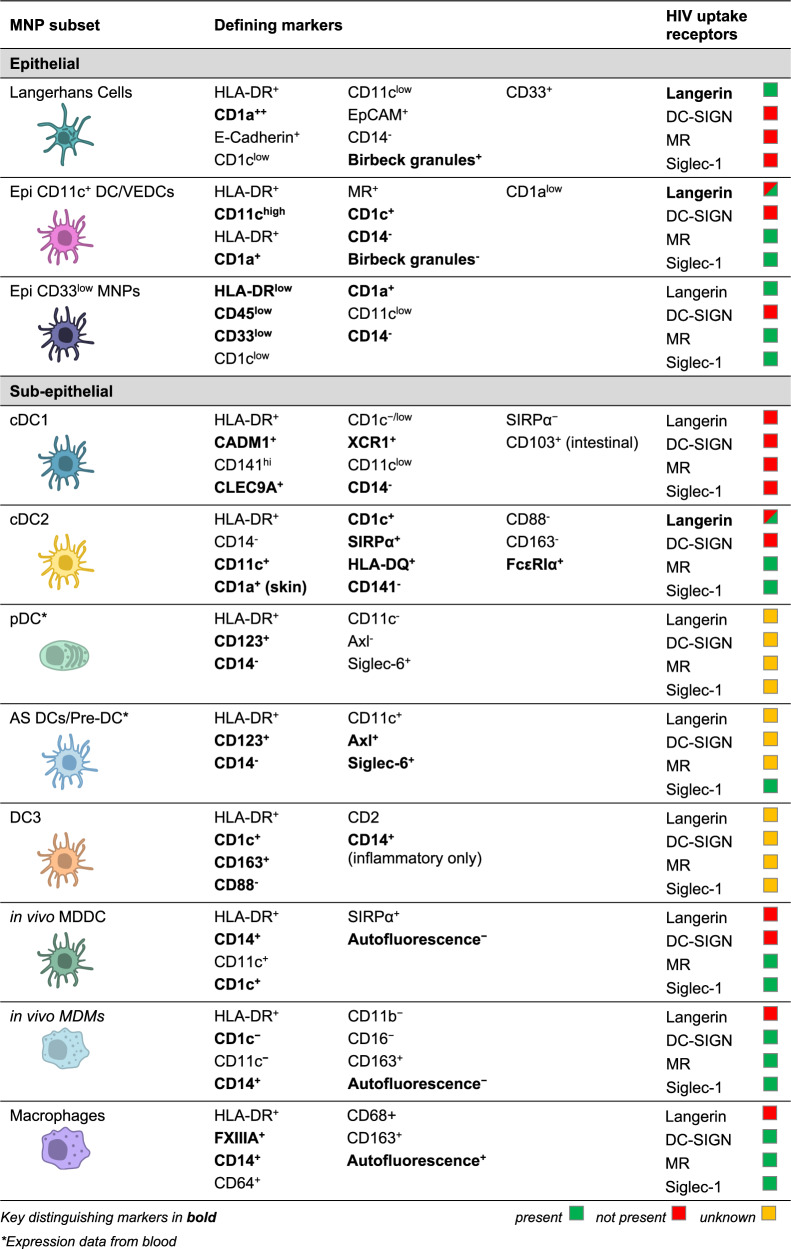
Human Mononuclear Phagocyte Phenotypes and their known HIV uptake receptors.

This review focusses on the functional role MNP play in sexual transmission of HIV which is highly dependent on their ability to capture the virus and then interact with CD4 T cells and transfer the virus to them. It is therefore the functional properties of these cells that will form the focus of our discussion.

### MNPs are key HIV transmitting target cells

In tissue, almost all MNPs express the HIV entry receptors CD4 and CCR5 as well as a range of HIV-binding lectin receptors. MNPs are therefore key HIV target cells. Importantly, binding of HIV to CD4/CCR5 leads to HIV infection of the cell whereas binding to some lectin receptors results in rapid uptake into neutral pH Virus Containing Compartments (VCCs) which are invaginations of the plasma membrane.^29–31^ This compartment is phenotypically identical to the VCCs created in infected macrophages, where they are believed to contribute to viral reservoirs in mucosal tissues.^32–34^ Connections that link the VCC to the cell surface can be very tight and therefore protect the virus from host immunity.^35–37^

Correlating with these two pathways of entry MNPs can transfer HIV to CD4 T cells in two successive phases.^31,38^ *Trans*-infection, (also called first-phase transfer) occurs when the MNP interacts with a CD4 T cell within 2–6 h, where virions are either held on the surface of the MNP^39,40^ or discharged from VCCs in a pulsatile fashion.^41^ This most likely occurs in the tissues where transmission takes place. After 6 h virions undergo acid proteolytic degradation over one or more days by a still undefined mechanism^41^ and by 24 h no transfer of HIV to CD4 T cells occurs. It is well known that LCs and DCs can perform this function but the capacity for macrophages to act in this capacity is still unclear. *Cis*-infection (also called second-phase transfer) occurs when a CD4 T cell interacts with MNPs 72 h or longer after the MNP has been infected via the CD4/CCR5 pathway, with the MNP actively producing virus that buds from the plasma membrane. This newly synthesised virus is transported to the tips of filopodia via actin. The filopodia contact target CD4 T cells and transfer HIV by subsequently forming a virological synapse.^42^ In addition, HIV-infected macrophages can induce tunnelling nanotubes creating a bridge between cells and allowing for the transfer of virus separate from the extracellular environment.^43^ *Cis*-infection increases with time as more virions are produced by the infected cell.

### HIV binding lectin receptors

MNPs express a wide range of lectin receptors on their surface which enable them to capture incoming pathogens. Each subset of MNP expresses a unique repertoire of these receptors meaning they differ in the specific pathogens they can capture. Langerin (CD207), DC-SIGN (CD209), Mannose Receptor (MR, CD206) and Siglec-1 (CD169), are currently the only MNP lectin receptors known to bind HIV.^44–46^ DC-SIGN was initially thought to be the most important but was then shown to be only expressed on MNPs along with MR in the dermis whereas langerin was expressed in the epidermis.^47^

**Langerin** is expressed most highly by LCs and has been extensively studied on authentic tissue-derived LCs and we refer the reader to the many reviews on this subject for a full historical perspective.^10,48–51^ HIV binds langerin in its trimeric form,^29^ which has been shown by some to lead to the internalisation of the virus into Birbeck granules where it is degraded which led to the hypothesis that langerin acts as a natural barrier to HIV infection.^52^ However, as discussed in detail below many others^53–55^ have shown that LCs can transfer HIV to CD4 T cells following langerin mediated uptake which can be blocked using soluble langerin or a langerin blocking monoclonal antibody.^29^

**DC-SIGN** was first described by Geijtenbeek et al. in 2000,^56–58^ and is the most extensively studied HIV binding lectin receptor. Using in vitro blood MDDCs, it has been shown to bind HIV in a tetrameric form^59^ and to play a role in the formation of the MHC-II-independent^60^ infectious synapse between DCs and T cells,^61,62^ whereby HIV is transferred to T cells in a protected environment while the cells are connected.^63^ Binding of HIV to DC-SIGN on these model cells triggers a signalling cascade that enhances *trans*-infection.^64^ Many studies have shown that tissue DC-SIGN expressing cells efficiently take up and transmit HIV.^65–68^

**MR** on in vitro blood MDDCs has been shown to bind HIV in a dimeric form^69^ and mediate endocytic uptake which leads to trafficking into lysosomes.^31^ MR expressing in vitro blood MDDCs have been shown to transmit HIV to CD4 T cells. However, it is not clear if this process was mediated by MR or via an alternate lectin receptor such as DC-SIGN which these cells also express.^31^

**Siglec-1** is the most recent lectin receptor implicated in HIV uptake and *trans*-infection, with the mechanism of the capture, internalisation and retention of exogenous virus in ‘vesicular caves’ seen on MNPs^70–73^ described before this interferon-inducible receptor was implicated.^72,74–77^ The formation of these caves sequesters the virus from the external environment, protecting it from neutralising antibodies and cellular immune responses, before it can be transferred to CD4 T cells. The cells’ ability to transfer HIV via these lectin-mediated cell-to-cell contacts may contribute to the evasion of HIV from neutralising antibodies and ART.^78^

These lectin receptors are found on a range of MNPs in transmission tissues, with differing expression levels (Table 1). The presence of these lectin receptors not only suggests their likely participation in HIV *trans*-infection, but also provides an additional means of MNP subset classification. Furthermore, the influence of receptor expression on HIV uptake by MNP subsets into VCCs, including DC versus macrophage, and retention versus degradation (thus determining the degree and kinetics of HIV transfer to T cells), needs to be carefully examined.

### Human tissue mononuclear phagocytes and HIV transmission

The continuously changing landscape of the MNP system can, at times, make it difficult to fully understand what specific subsets of MNP are being investigated and has likely contributed to conflicting findings in the HIV literature. For example, recent work indicates that epidermal DCs have been mis-defined. Most early studies of HIV and MNPs have made use of model in vitro derived MNPs and many studies still rely on these cells. These are most commonly derived in vitro from CD14^+^ blood monocytes to produce MDDCs or MDM. Alternatively CD34^+^ monocytes from cord blood can be used to generate monocyte-derived LCs (MDLC) and model LCs can also be derived from the MUTZ3 cell line.^79^ However, these model cells significantly differ from bona fide MNPs that reside within the anogenital tissue counterparts where transmission occurs. Notably, this particular model of MNP do not express the same repertoire of HIV binding lectin receptors as in vivo MNP. For example, in contrast to bona fide LCs, MDLCs express DC-SIGN and MR while in vitro MDDC and MDM do not express Siglec-1 in contrast to in vivo MDDC and MDM.^7,80^ In addition, compared to bone fide MNP, model MNPs express much higher levels of DC-SIGN and MR as well as the HIV entry receptors CD4 and CCR5. Caution must therefore be applied in interpreting the findings of these studies.^7^

## Epithelial HIV transmission

### Langerhans cells

Until very recently LCs were believed to be the sole MNP subset present within steady state stratified squamous epithelium that covers human skin and genital tissues. LCs are currently best defined by their high expression of HLA-DR, langerin and CD1a, low expression of CD11c, as well as the presence of distinct cytoplasmic structures known as Birbeck granules. They can be further distinguished from other MNPs by their lack of expression of MR, DC-SIGN and Siglec-1.^8,81^ Recent single cell transcriptomic analysis of the human stratified squamous epithelium has suggested that LCs exist as multiple subsets; two at steady state termed LC1 and LC2, an activated subset defined by high CD83 and low CCR7 expression termed LC3, and a migratory subset defined by high CCR7 expression termed LC4.^82^ Of the two steady-state LCs, LC1 were delineated as classic LCs while LC2 were described as a novel and unique subset of LC.

As LCs cells express the HIV entry receptor CD4^83–86^ and CCR5 in genital tissues and are found in closest proximity to the epithelial surface, they have been the most extensively studied MNP in the context of HIV transmission. Multiple early studies in the 1990s demonstrated that LCs within HIV exposed tissue contain HIV RNA^83,86,87^ and also the p24 protein.^84,88^ Furthermore, LCs were shown to efficiently transfer HIV to T cells using skin explant models^89,90^ and by co-culture of epidermal sheets with T cell lines.^91^ In 2007, Hladik et al. used human vaginal tissue explants to show that LCs rapidly took up HIV via endocytosis and were then able to migrate out of this tissue and interact with CD4 T cells. Importantly, HIV was shown to concentrate at the point of contact between LCs and CD4 T cells.^53^ In a similar study in 2010, Ganor et al. used human foreskin explants to show that LCs take up HIV within 1 h of exposure and then migrate to the basement membrane where they interact with and transfer to CD4 T cells.^54^ They then went on to show that this migration was meditated by RANTES.^92^ These studies support the hypothesis that LCs are able to capture HIV and then transmit the virus to CD4 T cells. However, in 2007 de Witte et al. published a landmark study that challenged this hypothesis and concluded that LCs in fact act as a natural barrier to HIV by showing that they efficiently take up HIV via langerin and traffic the virus to Birbeck granules where the virus become degraded.^93^ In 2014 Nasr et al. confirmed that LCs take up HIV via langerin by showing that both soluble langerin and a neutralising langerin antibody were able to block HIV uptake. However, in their hands LCs did not act as a natural barrier as they were able to efficiently transfer HIV to CD4 T cells within 2 h of exposure, while transfer was blocked by both the soluble langerin and the langerin blocking antibody. Furthermore, these LCs were also able to become productively infected and transfer the virus again at later time points.^29^ Though these findings may appear contradictory, it is important to note that de Witte et al., used trypsin to isolate DCs cleaving the binding site on CD4 for HIV and Nasr et al. could reproduce their negative findings with similar trypsin treatment. Furthermore, when de Witte et al. used higher concentrations of HIV (similar to that of Nasr et al.) they showed that this effect could be overcome. These higher concentrations of HIV may be physiologically relevant due to the high burst size of HIV from infected CD4 T cells^94^ that are present in semen and deposited on the anogenital mucosa during intercourse. Furthermore, amyloid fibrils in semen have been shown to increase the effective MOI of cell free virus by several orders of magnitude.^95^

### Conventional dendritic cell 2 (cDC2)—a newly identified HIV transmitting MNP

Misidentification of LCs in many of the studies described above may also explain the conflicting results on their role in HIV transmission. Recent studies have shown that LCs are not the only MNPs found in the stratified squamous epithelium in steady state. In 2018 Pena-Cruz et al. identified vaginal epithelial dendritic cells (VEDC).^96^ Like LCs, VEDCs expressed CD1a, langerin, CD4, CCR5 and not DC-SIGN. However, they did not express Birkbeck granules which are a defining feature of LCs. Concurrently, Bertram, Botting, Baharlou et al. identified epidermal CD11c^+^ DCs.^8^ These express the same markers as VEDC and can be discriminated from LCs by their (i) expression of MR, (ii) higher expression of CD11c, CD11b, CD1c, FcεR1α and HLA-DR and (iii) lower expression of langerin and CD1a. In abdominal tissue epidermal CD11c^+^ DCs are present in much lower proportions than LCs, but in foreskin they are present in roughly equal numbers and almost completely predominate over LCs in the epithelium of the vagina, fossa navicularis and anal canal.^8,96^ It is believed that VEDC/CD11c^+^ DCs were erroneously overlooked due to the method of isolation and their overlapping expression of key surface markers with LCs (HLA-DR, CD1a and langerin). Many groups (except Nasr et al.) liberated LCs from tissue exclusively using trypsin enzymatic digestion. However, Botting at al. showed that trypsin significantly cleaves the key identifying surface receptors expressed by these cells, CD11c and CD1c as well as the HIV entry receptor CD4.^8,81,96^ Therefore, unless CD11b, CD11c or CD1c are included as identification markers and trypsin is not used to liberate these cells from tissue, epidermal CD11c^+^ DCs/VEDCs cannot be reliably discerned from LCs. This means that LCs have almost certainly been misidentified in many studies, especially those examining these cells in anogenital tissues where CD11c^+^ DCs/VEDCs overwhelmingly predominate. Importantly, Bertram et al. showed that epidermal CD11c^+^ DCs were morphologically and transcriptionally undistinguishable from dermal CD11c^+^ conventional DC-2 (cDC2), suggesting these are in fact dermal cDC2 which have migrated into the stratified squamous epithelium. Functionally, however, epidermal CD11c^+^ DCs are much more efficient APCs compared to their dermal CD11c^+^ cDC2 counterparts. Furthermore, they secrete significantly higher levels of IL-1β, IL-6, IL-8, IL-10 and TNF than dermal CD11c^+^ cDC2 and do not secrete IL-1α.^8^ Furthermore, as described above, in 2021 Liu, Zhu et al. identified four subsets of LCs by analysing the transcriptional profile of the vaginal stratified squamous epithelium.^82^ The cells they denote as LC2 showed many phenotypic similarities to epidermal CD11c^+^ DCs in that they express the cDC2 specific transcription factor IRF4, lower levels of langerin and CD1a and higher levels of CD1c and CD11b. Furthermore, both cells are enriched in foreskin epidermis. However, unlike Bertram et al., they did not compare the transcriptional profiles of these cells to sub-epithelial MNPs or any other MNP, explaining why the similarity to cDC2 was not revealed. Finally, a similar cell has been observed in inflamed stratified squamous epithelium, termed inflammatory epidermal dendritic cells (IDEC).^97,98^ Similar to epidermal CD11c^+^ DCs, these cells express CD11c and MR. In conclusion, although further investigation is required, it is highly likely that epidermal CD11c^+^ DC, VEDC, LC2 and IDEC are one and the same cell, namely, sub-epithelial cDC2s that migrate into the stratified squamous epithelium, especially in the context of inflammation and in mucosal tissues which are rich in microbiota.

Importantly, both VEDCs and epidermal CD11c^+^ DCs are important cellular targets for HIV. Pena-Cruz et al. showed that VEDCs are highly permissive to infection by R5 HIV strains, whereas X4 viruses replicated inefficiently. Interestingly, however HIV binding and fusion was comparable for both R5 and X4 viruses, implying that the reduced replication was due to lower levels of integration and reverse transcription in addition to being influenced by restriction factor SAMHD1 which affects X4 viruses to a greater degree.^96^ Similarly, Bertram et al. showed that epidermal CD11c^+^ DCs take up HIV much more efficiently than LCs within 2 h. Importantly, using foreskin explant models and the in-situ hybridisation RNAscope technology, they were able to visualise these cells interacting with HIV within 30 min of topical exposure. Correlating with their high capacity to take up HIV and present antigen to CD4 T cells, these cells were also much more efficient than LCs at first phase (in trans) transfer of the virus to CD4 T cells within two hours. Furthermore, epidermal CD11c^+^ DCs expressed significantly higher levels of surface CCR5 than LCs and correspondingly supported higher levels of infection making them also more efficient than LCs at second phase (*in cis)* transfer of the virus to CD4 T cells at 72–96 h. Importantly second phase (*in cis)* transfer could be blocked with the CCR5-antagonist maraviroc confirming CD4/CCR5 meditated entry.^8^

Further demonstrating the importance of cDC2 in HIV transmission, Rhodes, Botting et al. recently demonstrated that sub-epithelial langerin expressing cDC2 are significantly more efficient at HIV uptake and transfer to CD4 T cells than their non-langerin expressing counterparts.^9^ As Bertram et al. showed that the majority of anogenital epidermal CD11c^+^ DC express langerin and transcriptionally align with cDC2, it is highly probable that sub-epithelial langerin^+^ cDC2 migrate to the epidermis where they capture HIV.^8^

## Sub-epithelial HIV transmission

Underlying the stratified squamous epithelial layer is a layer of sub-epithelial connective tissue referred to as dermis in skin (e.g. foreskin, anal verge, labia) and *lamina propria* in Type II mucosal tissue (vagina, fossa naviculars, anal canal, ectocervix). *Lamina propria* also underlies the Type I mucosa (penile urethra, rectum, endocervix) which has a thin and fragile epithelial surface monolayer consisting of columnar epithelial cells. As mucosal trauma is highly associated with HIV transmission, it is likely that HIV encounters the rich array of immune cells in these tissues which include multiple subsets of MNPs.

### The changing classification of CD14 + MNPs

In addition to CD11c^+^ cDC2s, the dermis and lamina propria also contain CD14 expressing MNPs which, in contrast to skin, represent the bulk MNP population in all anogenital and intestinal mucosal tissues.^7^ These cells were historically classified as either macrophages (defined by their autofluorescent properties and/or CD68 expression) or non-autofluorescent CD14^+^ DCs.^23^ In 2014, CD14^+^ DCs were redefined as a transient population of MDMs derived from blood CD14^+^ monocytes by McGovern, Schlitzer and colleagues.^24^ However, more recently Rhodes et al. showed that there are two populations of monocyte derived CD14^+^ cells, discerned by their expression of CD1c and CD11c.^7^ In agreement with McGovern et al., CD14^+^ CD1c^−^ CD11c^−^ cells were transcriptionally and morphologically macrophage-like and were also non-migratory and therefore defined as MDMs. Meanwhile, CD14^+^ CD1c^+^ CD11c^+^ cells were transcriptionally and morphologically DC-like and migrated out of tissue and therefore defined as MDDCs. Importantly, MDDCs do not express the key HIV binding lectin receptors DC-SIGN or langerin, and express very low levels of Siglec-1.^7^

### Re-evaluating the sub-epithelial MNP HIV transmission literature

In 2005, Gurney et al. demonstrated that DC-SIGN^+^ cells effectively captured and transferred HIV to CD4 T cells in the rectal mucosa. This viral capture was partially blocked by anti-DC-SIGN antibodies. These cells expressed CD14 and were defined as CD14^+^ DCs according to MNP definitions at this time. However, according to current definitions these cells are a heterologous population of yolk sac derived and monocyte-derived macrophages.^65^ Similarly, in 2012 Ganor et al. showed that HIV preferentially interacts with CD68^+^ resident macrophages in the penile urethra compared to T cells.^99^

In 2010, Shen, Smythies et al. showed that CD13 and CD11c expressing MNPs in the small intestine could capture HIV and transfer it in trans to CD4 T cells. This group went on to show similar findings in vaginal tissue.^100^ As CD14 and CD1c were not included as cell definition markers in either of these studies, these cells might consisted of a heterologous population of cDC2 and MDDC as well as of MDM as they were shown to express low levels of DC-SIGN.^101^ In 2013 Cavarelli et al. showed that DC-SIGN^+^ in vitro derived MDDCs sampled HIV in a tissue model. Although they extended this study using colonic tissue explants, DCs were defined as CD11c^+^ cells and thus, similar to the studies by Shen et al., these cells represented a heterologous population of cDC2 and MDDC.^66^

Three studies using human cervical tissue have demonstrated that MDDCs are preferential HIV target cells. In 2016, Rodriguez-Garcia et al. investigated HIV virus like particles capture on cervical CD11c^+^ cells^102^ and showed that HIV was exclusively captured by cells that co-expressed CD14, the majority of which did not express DC-SIGN. CD14^+^ CD11c^+^ DC-SIGN^−^ cells are currently defined as in vivo MDDCs. However, within this study a small proportion of HIV^+^ cells expressed DC-SIGN^+^ implying that a small proportion of MDMs were also present in this population. In 2018, Trifonova et al. confirmed these findings showing that cervical CD14^+^ CD11c^+^ MDDCs could take up HIV more efficiently than CD14^+^ CD11c^−^ MDM and CD4 T cells.^103^ The following year, Perez-Zsolt, Cantero-Pérez et al. showed that Siglec-1^+^ CD14^+^ CD11c^+^ cervical MDDCs could efficiently *trans*-infect CD4 T cells and that transfer was partially blocked by Siglec-1 antibodies.^104^

Most recently, in 2021 Rhodes et al. showed that sub-epithelial MDDCs could be key HIV target cells in the full range of human anogenital tissues. Although all CD14^+^ cells were able to take up HIV within 2 h, become productively infected and transfer the virus to CD4 T cells, MDDCs carried out these functions preferentially.^7^ Importantly, using RNAscope they visualised both CD14^+^ CD1c^+^ MDDC and macrophages interacting with the virus 2 h post treatment in foreskin and urethral explants. Consistent with Perez-Zsolt et al., using a Siglec-1 monoclonal antibody they could block up to 40% of HIV uptake on in vivo MDDCs, up to 60% on in vivo MDMs, and up to 85% on tissue-resident macrophages.^7^ The degree of blocking directly correlated to the expression of Siglec-1 on these MNP subsets, suggesting that Siglec-1 is a key receptor for HIV uptake on tissue MNPs.^7^

In conclusion, sub-epithelial tissues contain both macrophage and DC populations, most of which can capture HIV and can transfer the virus to CD4 T cells. However, after ex vivo infection of the sub-epithelial tissue compartment CD14^+^ DC-like cells are the likely predominant HIV transmitting population.

**Fig. 1:**
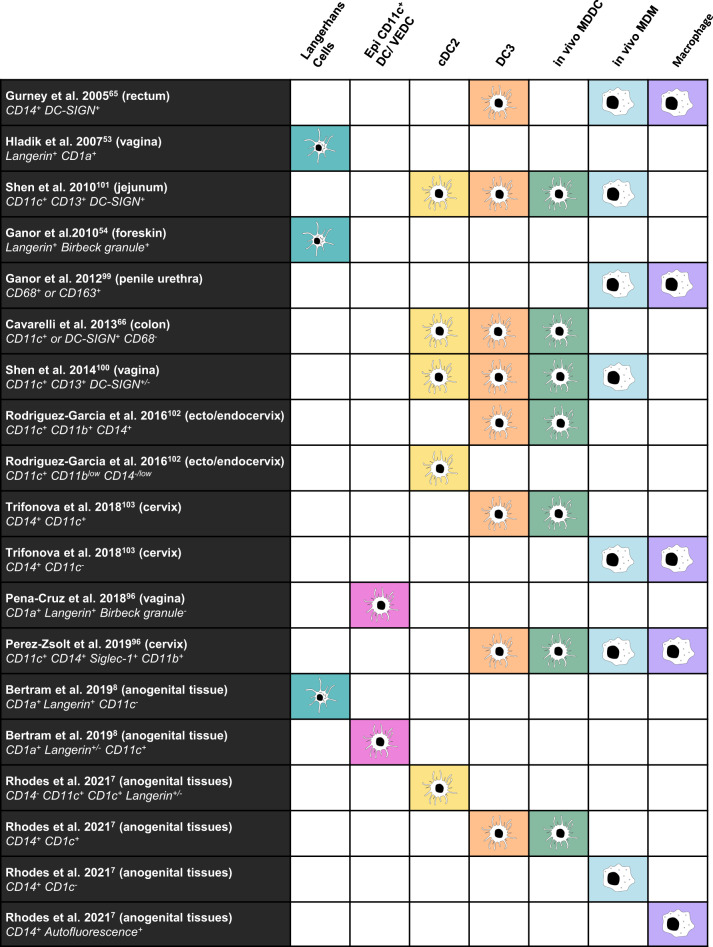
Current mononuclear phagocyte subsets represented in existing HIV literature. A selection of existing literature describing HIV interactions with transmission site mononuclear phagocytes were selected for re-examination. Based on the phenotypic data that was presented (left column lists the publications that were assessed, the tissue studied, and the defining phenotypic characteristics presented by the authors), key HIV transmitting mononuclear phagocytes from each study were categorised into the current mononuclear subsets (the columns on the right). This does not consider the proportional contribution that each subset represents, only whether each described mononuclear phagocyte matches the phenotypic profile of the current subset.

Figure 1 summarises the MNP subset(s) investigated in the above mentioned epithelial and sub-epithelial studies according to the most recent literature.

### Have macrophages been overlooked in HIV transmission?

Although macrophages have long been known to become infected by HIV, they have not been thought to play a role in HIV transmission as they are weak antigen presenting cells. Instead, they have been thought to form part of the HIV reservoir.^32^ However, it is important to draw attention to the fact that two high affinity HIV binding lectin receptors that were historically considered to be classical DC markers are now known to be macrophage specific: DC-SIGN and Siglec-1. As described in detail above, although DC-like MNPs are more efficient at HIV uptake, infection, and transfer of the virus to CD4 T cells, macrophages can also perform these functions. Furthermore, as demonstrated by Rhodes et al., macrophage-like cells are significantly more abundant than DC-like cells across all anogenital tissues.^7^ Therefore, while macrophages may not be as efficient at transmitting HIV, they may be no less important due to their high abundance.

## Future directions

### Is there a missing HIV binding lectin receptor on dendritic cells?

This review indicates that in human tissues there are three MNP subsets that are best at transmitting HIV at the anogenital sites: epidermal cDC2, sub-epithelial langerin^+^ cDC2 and MDDC. Of the currently known HIV binding lectin receptors (DC-SIGN, MR, langerin and Siglec-1) only MR is expressed by all three cell subsets. However, MR only weakly binds HIV and targets the virus for rapid proteolytic degradation rather than uptake into neutral pH VCCs.^31^ Similar to LCs, none of these cells express the high affinity HIV binding lectin DC-SIGN. MDDCs only express Siglec-1, but at very low levels and blocking Siglec-1 only very weakly inhibits HIV by these cells.^7^ Langerin^+^ cDC2 only express langerin, but at much lower levels than LCs which some groups have shown leads to viral degradation.^93^ Therefore, we propose that there is a yet to be identified HIV binding lectin receptor(s) expressed on the most efficient HIV transmitting DCs. Identification of this receptor(s) will have important implications for HIV blocking strategies and may also provide an avenue to direct HIV vaccine peptides to the actual antigen presenting cells that will most efficiently drive a targeted immune response to this virus.

### Inflammatory dendritic cells and HIV transmission

Although the subsets of MNPs that inhabit steady state human tissues are now well defined, those present in inflamed tissues still need to be delineated. This is a key gap in the literature as HIV transmission is now known to be strongly associated with inflammation.^105–107^ Concerningly, current PrEP regimens have been shown to be ineffective in the context of inflammation and anogenital inflammation is prevalent in sub-Saharan Africa where most acquisition of HIV still occurs. As cells migrate into inflamed tissues via CCR5 binding chemokine gradients, inflammatory cells therefore express higher levels of the HIV entry receptor CCR5, likely making them more permissive to HIV infection. This is evidenced by the recent findings by Liu et al. that LC2 are enriched in inflamed tissues^82^ and the findings by Bertram et al. who showed high levels of CCR5 on epidermal CD11c^+^ DCs^8^ which are almost certainly the same cells as LC2. In addition, unlike their steady-state counterparts, in vivo MDDCs express the HIV binding lectin DC-SIGN,^108^ likely enhancing their ability to take up HIV and transfer virus to CD4 T cells. Furthermore, the newly found inflammatory DC3s in blood have been shown to express high levels of DC-SIGN and langerin RNA, though this has not been investigated at the protein level or in tissue.^27^ Previously CD123^+^ BDCA2^+^ plasmacytoid DCs (pDCs) were the only known MNP cell type to exclusively inhabit inflamed tissues. Recently however, CD123^+^ BDCA2^+^ cells were shown to consist of a heterologous population of bona fide pDCs and Axl^+^ Siglec-6^+^ (AS) DCs^25^/pre-DC.^26,109^ In blood, AS DCs/pre-DCs have been shown to preferentially interact with HIV via Siglec-1,^110^ though this is yet to be investigated in inflamed tissue.

Therefore, as inflammatory MNP populations are better defined it is important to determine if these cells are present in the tissues where HIV transmission occurs and which HIV binding receptors they express. It will also be important to determine how efficiently they take up HIV, become infected, and transfer HIV to CD4 T cells. These findings will help in the development of modified PrEP strategies to block transmission of HIV in an inflamed setting. This could be particularly transformative for women in sub-Saharan Africa who are often disempowered to protect themselves.

## Concluding remarks

There are still 1.7 million new HIV infections each year and up to 1 million HIV-associated deaths. Furthermore, the cost of lifetime ART for all individuals in low- and middle-income countries is estimated to be $30 billion USD per year by 2030. Therefore, the need to block transmission of this virus remains a high global health priority. With the rapid advancement of high parameter single cell technologies our understanding of the MNP system will continue to evolve, especially in inflamed tissues. As our understanding deepens it is important that we translate these advancements to better define the role these cells play in HIV transmission. It is especially important that we accurately understand exactly which MNPs deliver HIV to CD4 T cells and the mechanism by which this occurs as this will guide designing vaccine strategies. Furthermore, prior to the development of an HIV vaccine this will also help in optimising PrEP regimens to block HIV acquisition.
